# Gender differences in sense of coherence among university students during the COVID-19 pandemic in Turkey

**DOI:** 10.1093/heapro/daad048

**Published:** 2023-06-03

**Authors:** Ilker Kayi, Gizem Uzunköprü, Kevin Dadaczynski, Pınar Soylar, Buğra Otludil, Pınar Dündar, Nadi Bakırcı, Orkan Okan, Sibel Sakarya

**Affiliations:** Department of Public Health, Koç University School of Medicine, İstanbul, Turkey; Koç University School of Medicine, İstanbul, Turkey; Department of Health Science, Fulda University of Applied Sciences, Fulda, Germany; Center for Applied Health Sciences, Leuphana University, Lueneburg, Germany; Department of Health Sciences, Nursing School, Fırat University, Elazığ, Turkey; Koç University School of Medicine, İstanbul, Turkey; Department of Public Health, Celal Bayar University School of Medicine, Manisa, Turkey; Acıbadem University School of Medicine, Dean, İstanbul, Turkey; Technical University Munich, TUM Department of Sports and Health Science, Munich, Germany; Department of Public Health, Koç University School of Medicine, İstanbul, Turkey

**Keywords:** gender, COVID-19, higher education, psychosomatic complaints, psychological well-being, sense of coherence

## Abstract

Non-pharmaceutical interventions (NPIs) implemented to mitigate the COVID-19 pandemic halted everyday life in higher education along with social and psychological impacts. The objective of our study was to explore the factors related to sense of coherence (SoC) from a gender perspective among university students in Turkey. This is a cross-sectional survey conducted online with a convenience sampling method as part of the international COVID-Health Literacy (COVID-HL) Consortium. SoC was measured by a nine-item questionnaire that was adapted to the Turkish language, including socio-demographic information and health status, including psychological well-being, psychosomatic complaints, and future anxiety (FA). 1595 students from four universities, of whom 72% were female, participated in the study. Cronbach’s alpha for the SoC scale was 0.75. Based on the median split of the individual scores, levels of SoC showed no statistically significant difference according to gender. Logistic regression analysis indicated that higher SoC was associated with medium and high subjective social status, studying in private universities, high psychological well-being, low FA, and none/one psychosomatic complaint. While results were similar among female students, type of university and psychological well-being showed no statistically significant association with SoC among males. Our results indicate that structural (subjective social status) and contextual (type of university) factors, along with gender-based variations, are associated with SoC among university students in Turkey.

## INTRODUCTION

Following the worldwide spread of a novel Coronavirus infection named the COVID-19 pandemic, the world stumbled into a social and economic shutdown. Due to the complex crisis in the health systems and the unavailability of any proven treatment or vaccines, countries have begun implementing non-pharmaceutical interventions (NPIs) such as physical distancing, mask-wearing, and stay-home orders, which restricted the everyday life activities of many people in order to halt or slow down the spread of the virus. Historically such measures might be successful tools for controlling an outbreak. However, these measures have the potential to put an extra burden on mental health due to the duration of isolation, fear of infection, stigma, frustration, boredom, inadequate information, financial consequences, and uncertainty in a continuing pandemic (Brooks *et al.*, 2020; Al Dhaheri *et al.*, 2021).

After the first COVID-19 case in Turkey on March 11th, 2020, pandemic control measures were put in place, including travel restrictions, stay-home recommendations, mandatory mask use, physical distancing, cancelation of outdoor activities, periodic partial lockdowns and closure of schools and universities ever since giving way to online learning activities (Koca, 2020). In addition, for age groups below 20 and over 65, the government declared a full or partial curfew between defined hours (Kanbur and Akgul, 2020). The most likely explanation for such an intervention was to protect the elderly who had a much higher mortality risk and to control the movements of young populations, who were presumed to be spreading the disease more likely in an asymptomatic or pre-symptomatic way (Kayi and Sakarya, 2020). However, the curfew, which lasted for more than six months, raised concerns regarding the mental health status of the affected groups.

Negative mental health experiences have not been rare among university students before the COVID-19 pandemic. According to the results of the World Mental Health Surveys International College Students Project conducted by the World Health Organization (WHO), one out of three first-year students experiences a mental health problem (Auerbach *et al.*, 2018). While NPIs restrained all people’s everyday interactions to control the COVID-19 pandemic, such targeted restrictions for youth have put an extra burden on the mental health status of university students. Thus, there have been efforts to investigate the mental health impacts of the COVID-19 pandemic on university students in Turkey. Available studies during the pandemic showed that students had increased anxiety, psychological distress, and dissatisfaction with life (Aslan *et al.*, 2020; Torun and Torun, 2020; Aslan and Pekince, 2021; Sogut *et al.*, 2021).

Under such health emergencies, applying the principles of the Salutogenic model and enhancing the sense of coherence (SoC) in university students have been suggested as a promising approach to promote their mental well-being (Dadaczynski *et al.*, 2021a). Salutogenesis focuses on the question of ‘what makes people healthy’ rather than on disease risk factors (Mittelmark and Bauer, 2017). SoC has a central position in the conceptualization of the model and is comprised of three core components: comprehensibility, manageability, and meaningfulness (Eriksson, 2017). According to Antonovsky (Antonovsky, 1987), comprehensibility refers to the cognitive perception of any stimuli, whether consistent and structured or random and chaotic to conceive the predictability of the situation people faced. While manageability refers to the perceived control over one’s resources and actions to cope with the stimuli one faces, meaningfulness refers to the emotional and cognitive perception of life challenges as worthy of commitment and dedication rather than a burden (Antonovsky, 1987; Eriksson and Mittelmark, 2017). Therefore, increased SoC has the potential to help overcome or endure the previously mentioned challenges arising from the uncertainties of the pandemic and restrictive pandemic measures such as physical distancing, mask-wearing, and stay-home orders. In a study from Italy conducted at the beginning of the pandemic, Barni *et al.* (Barni *et al.*, 2020) argue that besides fear, SoC, in terms of comprehensibility, can serve as a resource to cope with stressors and comply to the pandemic measures. Another study by Alegria *et al.* (Alegria *et al.*, 2021) found that a coherent understanding of COVID-19 risk reduced engaging in high-risk behaviors such as non-compliance with stay-home recommendations and increased healthy behaviors such as hand washing. Généreux *et al.* (Genereux *et al.*, 2021) indicate that efforts to increase SoC can support the adaptive capacities of individuals and groups to cope with adverse life experiences during the pandemic.

Antonovsky’s (Antonovsky, 1996) conceptualization of SoC depends on the life experiences of people, which are molded by position in the social structure, culture, gender, ethnicity, age, genetics, or other life chances. Gender has been one of the most studied factors in shaping the SoC, however, with mixed results (Volanen *et al.*, 2004; Natvig *et al.*, 2006; Hochwalder and Saied, 2018; Abu-Kaf and Khalaf, 2020; Kowalska *et al.*, 2020; Johansen *et al.*, 2021; Luibl *et al.*, 2021; Mana *et al.*, 2021a). In a systematic review by Rivera et al., gender differences have been considered a determining factor for SoC which has been reported higher among males in 12 of the 18 studies and no difference in the remaining six studies (Rivera *et al.*, 2013). Also, the lower socio-economic position has been shown to be associated with lower SoC levels (Feldt *et al*, 2005; Ristkari *et al.*, 2009; Silarova *et al.*, 2014). According to Dietscher et al., both gender and socio-economic position have a restricting role in the development of SoC (Dietscher *et al.*, 2017).

A strong SoC is a resource to promote health by developing effective coping mechanisms (Eriksson and Lindstrom, 2006). Studies are reporting the positive association between SoC and general perceived health (Henje Blom *et al.*, 2010; Moksnes *et al.*, 2011; Garcia-Moya *et al.*, 2013). Regarding mental health, SoC has been shown to be associated with increased resilience and emotional well-being (Braun-Lewensohn *et al.*, 2017). In their systematic review, Eriksson and Lindström (Eriksson and Lindstrom, 2005) has shown that SoC was strongly correlated with positive aspects of mental health such as self-esteem, self-efficacy, optimism, resourcefulness, and locus of control but negatively correlated with adverse outcomes of mental health such as anxiety, depression, feelings of anger and hostility, and perceived stressors. Such investigations have also been conducted in university settings. Darling et al. demonstrated that SoC was positively correlated with emotional health and quality of life and inversely correlated with stress from friendships and family issues among university students with distinct gender differences in the associations (Darling *et al.*, 2007). A recent study from China demonstrated that students with higher levels of SoC from a public university had significantly improved resilience against conduct problems, hyperactivity, peer problems, and emotional problems during the COVID-19 pandemic (Sun *et al.*, 2022).

University as an institution shifts the focus from the individual to the organization and facilitates dynamic interaction with the environment and community that can promote SoC within its structured and social system (Graeser, 2011). Dooris *et al.* (Dooris *et al.*, 2017) argue that higher education institutions serve as unique settings to strengthen the comprehensibility, manageability, and meaningfulness of their students to promote overall well-being through the multitude of opportunities provided by the universities for students to develop their capabilities to the highest level of their potential. In this study, we hypothesize that SoC will be distributed differently according to gender and socio-economic status. Previous studies on SoC in a university context have reported its association with gender. However, a gender-specific approach was lacking in most of the studies. In addition, based on the reports regarding the unequal impact of the COVID-19 pandemic on women (UN Women, 2020), our main objective was to investigate the factors, including gender and socio-economic status related to SoC among university students in Turkey during the COVID-19 pandemic.

## METHODS

### Study design and participants

This is a cross-sectional study conducted in Turkey with a convenience sampling approach. We have used a survey, which was launched in mid-March 2020 by an international COVID-Health Literacy (COVID-HL) Consortium (www.covid-hl.org) to investigate digital health literacy, sense of coherence, future anxiety (FA), and mental health outcomes among university students (Dadaczynski *et al.*, 2020).

A total of four universities (two private universities from Istanbul and two state universities from Manisa and Elazig) participated in the study. Data were collected from a convenience sample via an online survey between November and December 2020 within eight weeks. The office of student affairs in each participating university has sent the invitation letters for the online survey to all their students via email. After the initial invitation, three reminder emails were sent out at intervals of 2 weeks. Both graduate and undergraduate students over 18 years of age, who could read Turkish and were willing to participate in the survey were accepted into the study. Students from universities who did not participate in the project were excluded from the sample.

### Measurement

The survey consisted of three parts. The first part contained socio-demographic information such as gender (male versus female), age (open response), type of university (public vs. private school), department of study (health sciences, social sciences, and humanities, and science and engineering), level of study (undergraduate or graduate), number of semesters since enrollment, and subjective social status (SSS) assessed by the MacArthur Scale (Adler *et al.*, 2000; Hoebel *et al.*, 2015). Respondents were asked to position themselves on a hypothetical ladder of 10 steps that reflects their level of social status in their society. Steps 1–4 indicated low, 5–7 medium, and 8–10 the highest level in the social hierarchy (Dadaczynski *et al.*, 2021b).

The study’s dependent variable was SoC, assessed by the SoC scale initially developed for the work context (Vogt *et al.*, 2013). Therefore, verbal adaptations have been made by the COVID-HL Consortium to assess university students’ current living situation. The question ‘How do you personally find your current job responsibilities or work situation in general’ was amended as ‘How do you personally find your current life situation in general?’. The response items included nine bipolar adjectives with a seven-point differential scale from 0 to 6. The scale had three subscales, namely comprehensibility (four items: manageable, structured, clear, and predictable), manageability (two items: impossible to influence and controllable), and meaningfulness (three items: meaningful, significant, and rewarding). Overall, the reliability analysis of the scale yielded a Cronbach’s alpha of 0.83, along with the subscales that showed acceptable results with Cronbach’s alpha ranging between 0.72 and 0.84 (Dadaczynski *et al.*, 2021a). No changes have been made to the original response items due to their generic meanings.

The participants’ mental health was assessed with three variables in the last part of the survey. First, respondents were asked to respond to World Health Organization-5 (WHO-5) scale including five items using a six-point response option (0 = at no time, 1 = some of the time, 2 = less than half of the time, 3 = more than half of the time, 4 = most of the time, 5 = all of the time) about their psychological well-being during the last two weeks (Topp *et al.*, 2015). Eser *et al.* (Eser *et al.*, 2019) have adapted the original scale to Turkish and report the Cronbach’s alpha as 0.81. The total score of the WHO-5 scale ranged from 0 to 25, and this score was multiplied by 4 to obtain the final score. A participant’s psychological well-being was considered low when the score was ≤ 50. Second, psychosomatic complaints of participants were captured, assessing the frequency of certain symptoms such as headache, stomachache, backache, feeling low, irritability or bad temper, feeling nervous, difficulties in getting to sleep, and feeling dizzy in the previous six months (Haugland *et al.*, 2001; Hetland *et al.*, 2002). Each item was provided with a five-point response scale (0 = rarely or never, 1 = about every month, 2 = about every week, 3 = more than once a week, 4 = about every day). Haugland *et al.* (Haugland *et al.*, 2001) have reported the Cronbach’s alpha scores of the scale as 0.79. We grouped participants who reported two or more symptoms more than once a week or about every day as having multiple psychosomatic complaints (Inchley *et al.*, 2020). Third, we evaluated the students’ FA using a nine-item scale that illustrates the perceptions of dangers and adverse events people face in their personal lives in both local and global contexts. The first five items of the scale constitute the shorter version of the FA instrument (Zaleski, 1996) that was developed under the name of Dark Future Scale (Zaleski *et al.*, 2019). Additional four items were included to fit the purpose of the study from the prior scale version based on their face validity with some change in wording, for example, by replacing AIDS with COVID-19 and the Cronbach’s alpha score was obtained was 0.82 (Dadaczynski *et al.,* 2021a). The scale was rated by a Likert scale response option ranging from 0 (decidedly false) to 6 (decidedly true).

We translated the SoC and FA scales into Turkish. Forward-translation of the scales was completed by two bilingual members of the research team (G.U. and B.O.) independently. The translations were then compared by the whole research team to resolve the discrepancies and finalize the Turkish translation. The initial Turkish versions were back-translated to English by an independent translator. An expert committee of three public health professionals reviewed all versions of translated materials and approved the final versions of the scales by reaching a consensus on each item. Following the translation phase, debriefing interviews were held with ten university students as a pilot.

### Statistical analysis

Descriptive analyses were presented as percentages, mean, median and standard deviation. Age and number of semesters enrolled in the university were dichotomized according to their median value. Reliability analysis for the SoC scale was conducted by calculating Cronbach’s alpha values of the items for internal consistency. Cronbach’s alpha value of ≥ 0.7 was considered satisfactory (Bland and Altman, 1997). We split the SoC and FA scores based on the median value to dichotomize the variables as low (lower 50%) and high (upper 50%) levels of SoC and FA (Dadaczynski *et al.*, 2021a). Kolmogorov-Smirnov Test was performed to analyze the distribution pattern of SoC scores. We used Chi-Square Test for bivariate analysis and logistic regression analysis to investigate the association of selected socio-demographic characteristics and health status with dichotomized SoC variables. Both bivariate and multivariate analyses were run separately according to gender. Statistical analyses were performed by excluding the cases with missing data. Statistical significance was accepted as *p* < 0.05 in every analysis. All analyses were conducted using SPSS version 26.

## RESULTS

A total of 1684 students participated in the study. People who were not enrolled in a university program (*n* = 19) at either undergraduate level or graduate level, people who have started but have not completed the survey (*n* = 49), and people who have not provided gender information (*n* = 21) were excluded from the data. As a result, all analyses were conducted with a total of 1595 participants, of whom 72% were female. The mean age was 21.04 (SD = 3.17; median = 20). The length of the school enrollment measured by the number of semesters being a student in a university was 4.76 (SD = 4.37; median = 3), and one academic year consisted of three semesters.

Cronbach’s alpha measured for the internal consistency of the SoC and FA scales were 0.75 and 0.86, respectively. Detailed outcomes of reliability analysis can be found in Supplementary Tables 1 and 2. The mean score of the overall SoC scale was 29.38 (SD = 8.83, median = 29). Based on the recommended cut-off level, 33.7% of the students reported sufficient psychological well-being; and 63.8% reported two or more psychosomatic complaints (Table 1). While there was no statistically significant difference in SoC between male and female students (*p* = 0.291), 44.2% (*n* = 197) of male students reported sufficient levels of psychological well-being compared to 29.7% (*n* = 341) of female students (*p* < 0.001). Reporting no/one psychosomatic complaint was significantly more frequent among male students compared to female students (46.9% and 31.9%, respectively; *p* < 0.001), but lower levels of FA were significantly more common among female students compared to male students (47.7% and 58.3%, respectively; *p* < 0.001).

**Table 1:** Socio-demographic characteristics and health status of the participants according to gender

	Total *n* (%)	Female *n* (%)	Male *n* (%)	p
Age group				
≤ 20 years	804 (50.4)	611 (53.2)	193 (43.3)	<0.001
≥ 21 years	791 (49.6)	538 (46.8)	253 (56.7)	
Level of study				
Undergraduate	1437 (90.1)	1031 (89.7)	406 (91.0)	0.435
Graduate	158 (9.9)	118 (10.3)	40 (9.0)	
Department				
Health sciences	1004 (62.9)	783 (68.1)	221 (49.6)	<0.001
Social Sci. and Humanities	289 (18.2)	225 (19.6)	64 (14.3)	
Science and engineering	302 (18.9)	141 (12.3)	161 (36.1)	
University type				
Public	1200 (75.2)	850 (74.0)	350 (78.5)	0.062
Private	395 (24.8)	299 (26.0)	96 (21.5)	
Semesters spent in university				
≤ 3 semesters (1 year)	811 (50.8)	606 (52.7)	205 (46.0)	0.015
≥ 4 semesters	784 (49.2)	543 (47.3)	241 (54.0)	
Subjective social status				
Low	437 (27.4)	297 (25.8)	140 (31.4)	0.049
Medium	1022 (64.1)	757 (65.9)	265 (59.4)	
High	136 (8.5)	95 (8.3)	41 (9.2)	
Satisfaction with financial status				
Insufficient	847 (53.1)	597 (52.0)	250 (56.1)	0.141
Sufficient	748 (46.9)	552 (48.0)	196 (43.9)	
Chronic health condition				
No	1346 (84.4)	965 (84.0)	381 (85.4)	0.477
Yes	249 (15.6)	184 (16.0)	65 (14.6)	
Impairment				
No	1378 (86.4)	994 (86.5)	384 (86.1)	0.830
Yes	217 (13.6)	155 (13.5)	62 (13.9)	
Psychological well-being				
Low	1057 (66.3)	808 (70.3)	249 (55.8)	<0.001
Sufficient	538 (33.7)	341 (29.7)	197 (44.2)	
Psychosomatic complaints				
None or only one	576 (36.1)	367 (31.9)	209 (46.9)	<0.001
Two or more	1019 (63.9)	782 (68.1)	237 (53.1)	
Future anxiety level				
Low	808 (50.7)	548 (47.7)	260 (58.3)	<0.001
High	787 (49.3)	601 (52.3)	186 (41.7)	
Sense of coherence				
Low	867 (54.4)	634 (55.2)	233 (52.2)	0.291
High	728 (45.6)	515 (44.8)	213 (47.8)	

Gender differences in sense of coherence among university students during the COVID-19 pandemic in Turkey.

Table 2 shows the bivariate analysis of the association between SoC, socio-demographic characteristics, and health status. Among the socio-demographic variables, no statistically significant association existed between department, level of study, and SoC (*p* > 0.05). Students under 20 years of age had significantly higher SoC (*χ*^2^ = 10.373, *p* = 0.001), and this association remained significant both for females (*χ*^2^ = 4.651, *p* = 0.031) and males (*χ*^2^ = 8.049, *p* = 0.005). Similarly, students enrolled at the university for three semesters and less showed a significantly higher SoC among all students (*χ*^2^ = 14.476, *p* < 0.001), females (*χ*^2^ = 8.393, *p* = 0.004), and males (*χ*^2^ = 7.190, *p* = 0.007). While living with a chronic disease showed no significant association (*p* = 0.08), impairment due to any health problem was significantly associated with SoC (*χ*^2^ = 4.866, *p* = 0.027) among all students. No association was found between the level of study and SoC. However, SSS, satisfaction with financial status, psychological well-being, multiple psychosomatic complaints, and FA were significantly associated with SoC among females, males and all students alike (*p* < 0.05).

**Table 2:** Bivariate analysis of the association of SoC with demographic and socio-economic factors, and health status according to gender

	All students*			Female			Male		
	Low *n* (%)	High *n* (%)	*p*	Low *n* (%)	High *n* (%)	*p*	Low *n* (%)	High *n* (%)	*p*
Age									
≤ 20 years	405 (50.4)	399 (49.6)	0.001	319 (52.2)	292 (47.8)	0.031	86 (44.6)	107 (55.4)	0.005
≥ 21 years	462 (58.4)	329 (41.6)		315 (58.6)	223 (41.4)		147 (58.1)	106 (41.9)	
Department									
Health Sciences	542 (54.0)	462 (46.0)	0.381	424 (54.2)	359 (45.8)	0.415	118 (53.4)	103 (46.6)	0.828
Social Sci. and Humanities	167 (57.8)	122 (42.2)		133 (59.1)	92 (40.9)		34 (53.1)	30 (46.9)	
Science and Engineering	158 (52.3)	144 (47.7)		77 (54.6)	64 (45.4)		81 (50.3)	80 (49.7)	
Level of study									
Undergraduate	779 (54.2)	658 (45.8)	0.722	569 (55.2)	462 (44.8)	0.983	210 (51.7)	462 (44.8)	0.485
Graduate	88 (55.7)	70 (44.3)		65 (55.1)	53 (44.9)		23 (57.5)	17 (42.5)	
Length of enrollment in the university									
≤ 3 semesters	403 (49.7)	408 (50.3)	<0.001	310 (51.2)	296 (48.8)	0.004	93 (45.4)	112 (54.6)	0.007
≥ 4 semesters	464 (59.2)	320 (40.8)		324 (59.7)	219 (40.3)		140 (58.1)	101 (41.9)	
University type									
Public	701 (58.4)	499 (41.6)	<0.001	509 (59.9)	341 (40.1)	<0.001	192 (54.9)	158 (45.1)	0.035
Private	166 (42.0)	229 (58.0)		125 (41.8)	174 (58.2)		41 (42.7)	55 (57.3)	
Subjective social status									
Low	308 (70.5)	129 (29.5)	<0.001	214 (72.1)	83 (27.9)	<0.001	94 (67.1)	46 (32.9)	<0.001
Medium	519 (50.8)	503 (49.2)		391 (51.7)	366 (48.3)		128 (48.3)	137 (51.7)	
High	40 (29.4)	96 (70.6)		29 (30.5)	66 (69.5)		11 (26.8)	30 (73.2)	
Satisfaction with financial status									
Insufficient	557 (65.8)	290 (34.2)	<0.001	403 (67.5)	194 (32.5)	<0.001	154 (61.6)	96 (38.4)	<0.001
Sufficient	310 (41.4)	438 (58.6)		231 (41.8)	321 (58.2)		79 (40.3)	117 (59.7)	
Chronic disease									
No	719 (53.4)	627 (46.6)	0.080	524 (54.3)	441 (45.7)	0.171	195 (51.2)	186 (48.8)	0.277
Yes	148 (59.4)	101 (40.6)		110 (59.8)	74 (40.2)		38 (58.5)	27 (41.5)	
Impairment									
No	734 (53.3)	644 (46.7)	0.027	540 (54.3)	454 (45.7)	0.141	194 (50.5)	190 (49.5)	0.07
Yes	133 (61.3)	84 (38.7)		94 (60.6)	61 (39.4)		39 (62.9)	23 (37.1)	
Psychological well-being									
Low	673 (63.7)	384 (36.3)	<0.001	518 (64.1)	290 (35.9)	<0.001	155 (62.2)	94 (37.8)	<0.001
High	194 (36.1)	344 (63.9)		116 (34.0)	225 (66.0)		78 (39.6)	119 (60.4)	
Psychosomatic complaints									
No	234 (40.6)	342 (59.4)	<0.001	150 (40.9)	217 (59.1)	<0.001	84 (40.2)	125 (59.8)	<0.001
Yes	633 (62.1)	386 (37.9)		484 (61.9)	298 (38.1)		149 (62.9)	88 (37.1)	
Future anxiety									
Low	328 (40.6)	480 (59.4)	<0.001	226 (41.2)	322 (58.8)	<0.001	102 (39.2)	158 (60.8)	<0.001
High	539 (68.5)	248 (31.5)		408 (67.9)	193 (32.1)		131 (70.4)	55 (29.6)	

Gender differences in sense of coherence among university students during the COVID-19 pandemic in Turkey.

In the last analytical step, the association of selected variables from bivariate analysis with SoC was assessed in multivariate analysis (Table 3). The selection was made based on the statistical significance found in the bivariate analyses. Among two variables that might serve as a potential collinearity between each other, we have selected length of university enrollment over age and SSS over financial satisfaction because the length of enrollment in university allows us to differentiate the exposure to university context better than age. SSS includes a perception of income along with education and occupation to represent the living standards of a person via the assessment of their perceived position in the social hierarchy (Giatti *et al.*, 2012). Gender was included in the all-student model as a socio-demographic variable of concern. According to the results among all students, there was no statistically significant association between gender (OR = 1.057; 95% CI = 0.827–1.351), impairment (OR = 1.171; 95% CI = 0.850–1.613), and SoC. According to the logistic regression analysis conducted within gender categories, being a student in a private university (OR = 1.841; 95% CI 1.362–2.488) and reporting a sufficient level of psychological well-being (OR = 2.208; 95% CI 1.632–2.988) were found to be associated with higher SoC level only among females. Having multiple psychosomatic complaints and FA is statistically significantly associated with lower SoC among both females (OR = 1.457; 95% CI = 1.086–1.954 and OR = 2.074; 95% CI = 1.579–2.724 respectively) and males (OR:1.783; 95% CI = 1.145–2.778 and OR:2.737; 95% CI = 1.773–4.225 respectively) and all students (OR:1.531; 95% CI = 1.200–1.953 and OR:2.235; 95% CI = 1.777–2.813, respectively).

**Table 3:** Multivariate analysis of SoC according to gender

	All students^*^		Female		Male	
	OR	95% CI	OR	95% CI	OR	95% CI
Length of enrollment in a university program						
≤ 3 semesters	1.335	1.072–1.661	1.280	0.988–1.659	1.506	0.995–2.279
≥ 4 semesters	R	–	R	–	R	–
Type of university						
Public	R	–	R	–	R	–
Private	1.670	1.288–2.165	1.841	1.362–2.488	1.265	0.754–2.123
Subjective social status						
Low	R	–	R	–	R	–
Med.	1.699	1.314–2.198	1.719	1.261–2.344	1.689	1.057–2.699
High	3.650	2.314–5.758	3.430	1.987–5.919	4.538	1.941–10.608
Impairment						
No	1.171	0.850–1.613	1.251	0.858–1.824	1.005	0.540–1.871
Yes	R	–	R	–	R	–
Psychological well-being						
Very low and low	R	–	R	–	R	–
Sufficient	1.939	1.510–2.489	2.208	1.632–2.988	1.450	0.922–2.279
Psychosomatic complaints						
None/One	1.531	1.200–1.953	1.457	1.086–1.954	1.783	1.145–2.778
Two or more	R	–	R	–	R	–
Future anxiety						
Low	2.235	1.777–2.813	2.074	1.579–2.724	2.737	1.773–4.225
High	R	–	R	–	R	–

OR, Odds Ratio; CI, Confidence Interval.

^*^Logistic regression analysis was adjusted according to gender.

## DISCUSSION

As part of the COVID-HL Consortium, we aimed to investigate the association of socio-demographic characteristics and mental health outcomes with SoC among university students during the pandemic in Turkey with a focus on gender differences. Our study indicated no significant association between gender and SoC in both bivariate and multivariate analyses. Other studies have shown similar findings (Volanen *et al.*, 2004, 2007; Hochwalder and Saied, 2018). Studies conducted in Turkey before to the pandemic also showed no gender difference in SoC in university students (Öztekin, 2009; Sezgin and Arıcı Doğan, 2021). However, being a private university student, with less than 3 semesters of enrollment (one academic year), medium or high SSS, sufficient psychological well-being, up to one psychosomatic complaint, and low FA have all been associated with higher SoC among all students.

According to Antonovsky, SoC was expected to stabilize after the age of 30 except for some minor modifications that can occur throughout adulthood (Antonovsky, 1987; Antonovsky *et al.*, 1990). However, longitudinal studies have yielded counter-results to Antonovsky’s hypothesis. In a Swedish cohort of 43 500 people aged 18–85, Nilsson *et al.* (Nilsson *et al.*, 2010) have found that SoC increases with age. A similar finding by Feldt *et al.* (Feldt *et al.*, 2011) among a Finnish cohort established an increase in SoC after a 5-year follow-up. In a study from Turkey, Sezgin and Arıcı-Doğan (2021) found no significant age differences in SoC in university students. Contrary to the literature, we found that high SoC was significantly associated with younger age (≤20 years) or less than 3 years of enrollment in university in the whole sample. In other words, first-year students’ SoC levels were higher than senior students. Therefore, lower SoC among senior students might be due to the disrupted routine of everyday life during the academic year compared to previous years, including various aspects of campus and city life. An analysis by Douglas *et al.* (Douglas *et al.*, 2020) suggests that young people whose education process was disrupted and who face unemployment risk in the long term are more vulnerable to the negative impacts of the pandemic. For the first-year students in Turkey, who have proceeded towards their higher education after being successful in the university entrance exams, SoC might be higher due to progress rather than disruption. Another explanation might be the COVID-19 pandemic itself that has potentially caused a decline in the level of SoC among senior students rather than the first-year students due to approaching graduation in the future under pandemic conditions. Supporting evidence for this is the unpublished analysis in our study that indicated that FA was significantly higher among students enrolled for four semesters and more.

Sezgin and Arıcı Doğan (2021) have used parental education levels as representative of socio-economic variables in their study among university students in Turkey and have shown no significant association between mother’s and father’s educational attainment and SoC. Our study used the type of university and SSS as a socio-economic indicator, both of which were significantly associated with SoC among all students. While being a student in a private university was significantly associated with high SoC among only female students, the distribution of SoC levels was not equal in terms of SSS regardless of gender in favor of the students with medium or high SSS. Dadaczynski *et al.* (Dadaczynski *et al.*, 2021a) have also shown that SoC was unequally distributed according to the social background of university students in their study in Germany. Similarly, previous studies on SoC show an association between SoC and socio-economic status (Silva *et al.*, 2021) as well as income levels (Ing and Linda, 2003; Zilinskas *et al.*, 2021). All students in our study reporting high and medium SSS regardless of gender might have been enjoying the resources available to them by their socio-economic advantage in the social hierarchy, providing opportunities that can make life experiences more comprehensible and manageable, as Ing and Reutler indicated (Ing and Linda, 2003).

Another indicator of socio-economic difference we used was the type of university, and it was not associated with SoC among male students. However, female students in private universities had significantly higher levels of SoC in our study. We believe that our findings represent gender inequality in universities in Turkey. In a report evaluating gender equality in higher education institutions in Turkey, private universities have been found to be more gender equal compared to public universities (Gender and Women’s Studies Research Center, 2019). Therefore, female students might have been experiencing the positive outcomes of more gender-inclusive environment in private universities that contribute to the SoC; however, the characteristics of the university environment were out of the scope of this study.

In a study before the pandemic in Turkey, Yalnizca-Yildirim and Cenkseven-Onder (2022) have shown that SoC significantly correlated with subjective well-being, life satisfaction, and positive affect in a positive direction. Similarly, Oztekin and Tezer (2009) have demonstrated that SoC had a predictive role in positive affect on students from a university in Turkey. The COVID-19 pandemic created a mentally stressful process for university students in many countries, but it has put an extra burden on people under the age of 20 in Turkey due to special curfew order applied to these groups as part of the NPIs. Shafer *et al.* (Schafer *et al.*, 2020) have demonstrated that higher levels of pre-pandemic SoC have been preventive for worsening of mental health status, and it has been expressed that the experience of the NPIs, such as stay-home orders or travel restrictions, have not been the same for both sexes. Women have been reported to be disproportionately affected by the indirect impacts of the pandemic responses resulting in increased violence, cramped living conditions, and barriers to accessing health care (UN Women, 2020; Fisseha *et al.*, 2021) with a consequence of exacerbated mental health problems (Almeida *et al.*, 2020). Our results indicate that compared to their male counterparts, female students’ psychological well-being was lower, along with significantly more psychosomatic complaints and higher FA levels. These findings are in line with previous comparisons between male and female students (Bayram and Bilgel, 2008; Torun and Torun, 2020; Silva *et al.*, 2021) regarding mental health. Also, high SoC was significantly associated with a sufficient level of psychological well-being, none/one psychosomatic complaint, and low FA among all students, which was in line with the available literature. Mana *et al.* (Mana *et al.*, 2021b) have shown a strong association between SoC and psychological well-being during the COVID-19 pandemic among seven countries despite their socio-cultural differences. In two recent studies, Dodd *et al.* (Dodd *et al.*, 2021) found a significant association between SoC and well-being among university students in Australia, and Dadaczynski *et al.* (Dadaczynski *et al.*, 2021a) have illustrated the association of the subdimensions of SoC, namely comprehensibility, meaningfulness, and manageability with psychological well-being.

In our study, while none/one psychosomatic complaint and lower levels of FA showed significant associations with higher SoC among female and male students alike, there was a gender difference in psychological well-being showing a significant association with SoC only among female students. Such a gender difference might be because of the varying degrees of significance in the association between the dimensions of SoC (comprehensibility, manageability, and meaningfulness) and psychological well-being. Supplementary Tables 3–5 illustrate that only the manageability dimension was not significantly associated with psychological well-being among males, which has the potential to influence the association between overall SoC and psychological well-being.

### Limitations

The cross-sectional nature of our study does not allow us to interpret the causal relationship between psychological well-being, psychosomatic complaints, and FA. Also, we had a convenience sample that limited the generalizability of our results. However, one of the strengths of our study is the high number of participants from different parts of Turkey, which allowed us to obtain significant results. Among these participants, students from health sciences had a higher proportion which might have influenced our results to report increased mental health outcomes, especially during the COVID-19 pandemic (Torun and Torun, 2020). Also, the timing of the study has been discussed to be a potential bias as SoC is viewed as life orientation in the current living circumstances (Eriksson and Lindstrom, 2006). Exams have been shown to be one crucial factor associated with a decrease in the SoC among students (Cohen *et al.*, 2008). However, the date interval of our data collection corresponded to the period between midterms and the final examination, therefore, any pressure related with the exams can be viewed as minimal. Lastly, similar to the study by Dodd *et al.* (Dodd *et al.*, 2021), we have reported the overall SoC levels rather than SoC subscales in our study due to the low Cronbach’s Alpha score of the manageability dimension. Thus, the interpretation of the results should be made with caution.

## CONCLUSION

Our findings support the idea that SoC is closely associated with everyday mental health outcomes, especially under stressful pandemic conditions. Investing in the development of SoC in students as part of the standard curricula in the university can pay off in better coping during public health emergencies. The association between SoC and mental health variables in our data (psychological well-being, FA, psychosomatic complaints) allows us to underline the recommendation by Hochwalder and Saied (Hochwalder and Saied, 2018) that SoC can be used as a screening tool to detect psychologically vulnerable groups. It is also important to underline that along with psychological factors, there are structural (social status) and contextual (type of university) factors in play to shape the SoC (perceptions of comprehensibility, manageability, and meaningfulness) among university students with varying weight according to gender. Therefore, we encourage further studies to investigate and develop SoC among university students adopting a healthy setting approach focusing on gender differences and socio-economically disadvantaged groups to ensure equity.

## Supplementary Material

daad048_suppl_Supplementary_Tables
