# Supplementary material for: Gender differences in sense of coherence among university students during the COVID-19 pandemic in Turkey
Source: Health Promot Int. 2023 Jun 3;38(3):daad048. doi: 10.1093/heapro/daad048 (PMC10243759; doi:10.1093/heapro/daad048)
Supplement: daad048_suppl_Supplementary_Tables [file daad048_suppl_supplementary_tables.docx]

**Gender Differences in Sense of Coherence among University Students During the COVID-19 Pandemic in Turkey**

**SUPPLEMENTARY TABLES**

**Supplementary Table 1.** Reliability analysis of Future Anxiety Scale

| **Scale item** | **Scale Mean if**  **Item Deleted** | **Scale Variance if**  **Item Deleted** | **Corrected Item-Total**  **Correlation** | **Squared Multiple**  **Correlation** | **Cronbach's Alpha if**  **Item Deleted** |
| --- | --- | --- | --- | --- | --- |
| FA 1 | 283.731 | 111.755 | 0.624 | 0.426 | 0.845 |
| FA 2 | 286.318 | 110.655 | 0.617 | 0.437 | 0.846 |
| FA 3 | 287.488 | 104.225 | 0.746 | 0.593 | 0.832 |
| FA 4 | 276.250 | 113.244 | 0.599 | 0.403 | 0.848 |
| FA 5 | 277.766 | 109.189 | 0.681 | 0.501 | 0.840 |
| FA 6 | 286.411 | 106.587 | 0.687 | 0.532 | 0.839 |
| FA 7 | 296.566 | 123.791 | 0.380 | 0.224 | 0.865 |
| FA 8 | 290.173 | 109.901 | 0.629 | 0.402 | 0.845 |
| FA 9 | 283.119 | 118.861 | 0.367 | 0.199 | 0.871 |

**Supplementary Table 2.** Reliability analysis of Sense of Coherence Scale

| **Scale item** | **Scale Mean if**  **Item Deleted** | **Scale Variance if**  **Item Deleted** | **Corrected Item-Total**  **Correlation** | **Squared Multiple**  **Correlation** | **Cronbach's Alpha if**  **Item Deleted** |
| --- | --- | --- | --- | --- | --- |
| SoC 1 | 256.052 | 64.554 | 0.392 | 0.184 | 0.729 |
| SoC 2 | 260.545 | 61.097 | 0.481 | 0.342 | 0.714 |
| SoC 3 | 261.021 | 62.154 | 0.453 | 0.248 | 0.719 |
| SoC 4 | 263.929 | 70.956 | 0.156 | 0.086 | 0.763 |
| SoC 5 | 255.557 | 61.463 | 0.492 | 0.349 | 0.712 |
| SoC 6 | 263.175 | 60.000 | 0.533 | 0.345 | 0.705 |
| SoC 7 | 260.817 | 66.352 | 0.356 | 0.254 | 0.734 |
| SoC 8 | 264.604 | 60.791 | 0.509 | 0.334 | 0.709 |
| SoC 9 | 263.063 | 63.463 | 0.430 | 0.278 | 0.723 |

**Supplementary Table 3.** Multivariate analysis of Comprehensibility according to gender

|  |  | **Female** | | **Male** | |
| --- | --- | --- | --- | --- | --- |
|  |  | **OR** | **95% CI** | **OR** | **95% CI** |
| **Length of enrollment in a university program** | ≤ 3 semesters | 1.239 | 0.965-1.591 | 1.442 | 0.972-2.138 |
|  | ≥ 4 semesters | R | -- | R | -- |
| **Type of University** | Public | R | -- | R | -- |
|  | Private | 1.378 | 1.031-1.842 | 1.318 | 0.808-2.148 |
| **Subjective SES** | Low | R | -- | R | -- |
|  | Med. | 1.590 | 1.181-2.142 | 1.645 | 1.051-2.573 |
|  | High | 2.840 | 1.685-4.787 | 2.516 | 1.169-5.416 |
| **Impairment** | No | 1.344 | 0.933-1.936 | 1.033 | 0.573-1.864 |
|  | Yes | R | -- | R | -- |
| **Psychological Well-being** | Low | R | -- | R | -- |
|  | High | 2.186 | 1.627-2.935 | 1.763 | 1.139-2.729 |
| **Multiple psychosomatic Complaints** | None/One | 1.262 | 0.949-1.680 | 1.080 | 0.703-1.659 |
|  | Two or more | R | -- | R | -- |
| **Future anxiety** | Low | 1.371 | 1.050-1.790 | 1.409 | 0.928-2.137 |
|  | High | R | -- | R | -- |

**Supplementary Table 4.** Multivariate analysis of Manageability according to gender

|  |  | **Female** | | **Male** | |
| --- | --- | --- | --- | --- | --- |
|  |  | **OR** | **95% CI** | **OR** | **95% CI** |
| **Length of enrollment in a university program** | ≤ 3 semesters | 1.185 | 0.925-1.518 | .888 | 0.601-1.311 |
|  | ≥ 4 semesters | R | -- | R | -- |
| **Type of University** | Public | R | -- | R | -- |
|  | Private | 1.030 | 0.772-1.373 | 1.225 | 0.756-1.985 |
| **Subjective SES** | Low | R | -- | R | -- |
|  | Med. | 1.286 | 0.956-1.730 | .919 | 0.591-1.428 |
|  | High | 1.556 | 0.941-2.572 | 1.367 | 0.649-2.880 |
| **Impairment** | No | 1.088 | 0.759-1.558 | .931 | 0.523-1.656 |
|  | Yes | R | -- | R | -- |
| **Psychological Well-being** | Low | R | -- | R | -- |
|  | High | 1.493 | 1.117-1.996 | 1.327 | 0.863-2.043 |
| **Multiple psychosomatic Complaints** | None/One | 1.119 | 0.844-1.485 | 1.081 | 0.707-1.653 |
|  | Two or more | R | -- | R | -- |
| **Future anxiety** | Low | 1.609 | 1.234-2.099 | 1.870 | 1.235-2.833 |
|  | High | R | -- | R | -- |

**Supplementary Table 5.** Multivariate analysis of Meaningfulness according to gender

|  |  | **Female** | | **Male** | |
| --- | --- | --- | --- | --- | --- |
|  |  | **OR** | **95% CI** | **OR** | **95% CI** |
| **Length of enrollment in a university program** | ≤ 3 semesters | 1.270 | 0.983-1.640 | 1.546 | 1.020-2.342 |
|  | ≥ 4 semesters | R | -- | R | -- |
| **Type of University** | Public | R | -- | R | -- |
|  | Private | 1.730 | 1.282-2.334 | 1.067 | 0.637-1.784 |
| **Subjective SES** | Low | R | -- | R | -- |
|  | Med. | 1.794 | 1.326-2.426 | 1.742 | 1.081-2.807 |
|  | High | 3.019 | 1.758-5.184 | 3.456 | 1.533-7.792 |
| **Impairment** | No | 1.185 | 0.820-1.714 | .674 | 0.361-1.257 |
|  | Yes | R | -- | R | -- |
| **Psychological Well-being** | Low | R | -- | R | -- |
|  | High | 2.433 | 1.794-3.297 | 1.660 | 1.057-2.607 |
| **Multiple psychosomatic Complaints** | None/One | 1.476 | 1.102-1.977 | 1.997 | 1.282-3.110 |
|  | Two or more | R | -- | R | -- |
| **Future anxiety** | Low | 1.709 | 1.305-2.237 | 2.504 | 1.615-3.882 |
|  | High | R | -- | R | -- |
